# The Role of Cell-Free DNA in Cancer Treatment Decision Making

**DOI:** 10.3390/cancers14246115

**Published:** 2022-12-12

**Authors:** András Telekes, Anna Horváth

**Affiliations:** 1 Omnimed-Etosz, Ltd., 81 Széher Rd., 1021 Budapest, Hungary; 2 Semmelweis University, 26. Üllői Rd., 1085 Budapest, Hungary; 3 Department of Internal Medicine and Haematology, Semmelweis University, 46. Szentkirályi Rd., 1088 Budapest, Hungary

**Keywords:** circulating tumor DNA (ctDNA), cell-free DNA (cfDNA), solid tumor, clinical decision making

## Abstract

**Simple Summary:**

The aim of this review is to evaluate the present status of the use of cell-free DNA and its fraction of circulating tumor DNA (ctDNA) because this year July 2022, an ESMO guideline was published regarding the application of ctDNA in patient care. In the near future the data obtained from ctDNA may routinely be used for finding minimal residual disease, detecting relapse, determining either the unknown primary tumor, or the site of metastases. It can also be used for deciding upon the appropriate efficiency of the therapy and/or emerging resistance to the therapy. Therefore, clinicians should be aware of the potentials and the limitations of the assays. Of course, several open questions are still under research and as a result, cfDNA and ctDNA testing are not part of routine care yet.

**Abstract:**

The aim of this review is to evaluate the present status of the use of cell-free DNA and its fraction of circulating tumor DNA (ctDNA) because this year July 2022, an ESMO guideline was published regarding the application of ctDNA in patient care. This review is for clinical oncologists to explain the concept, the terms used, the pros and cons of ctDNA; thus, the technical aspects of the different platforms are not reviewed in detail, but we try to help in navigating the current knowledge in liquid biopsy. Since the validated and adequately sensitive ctDNA assays have utility in identifying actionable mutations to direct targeted therapy, ctDNA may be used for this soon in routine clinical practice and in other different areas as well. The cfDNA fragments can be obtained by liquid biopsy and can be used for diagnosis, prognosis, and selecting among treatment options in cancer patients. A great proportion of cfDNA comes from normal cells of the body or from food uptake. Only a small part (<1%) of it is related to tumors, originating from primary tumors, metastatic sites, or circulating tumor cells (CTCs). Soon the data obtained from ctDNA may routinely be used for finding minimal residual disease, detecting relapse, and determining the sites of metastases. It might also be used for deciding appropriate therapy, and/or emerging resistance to the therapy and the data analysis of ctDNA may be combined with imaging or other markers. However, to achieve this goal, further clinical validations are inevitable. As a result, clinicians should be aware of the limitations of the assays. Of course, several open questions are still under research and because of it cfDNA and ctDNA testing are not part of routine care yet.

## 1. Introduction

Diagnoses and treatment decisions in oncology are based upon histological investigation and sometimes molecular profiling of tissue samples. The latter is gaining more and more importance when selecting appropriate treatment for individual patients. Moreover, in the case of metastatic disease, the treatments are targeted to the metastases instead of the primary tumor, if possible. Consequently, the role of liquid biopsy in decision making is under investigation regarding circulating tumor cells [1] (CTC) as well as cell-free (or circulating free) DNA (cfDNA) [2]. The majority of cfDNA originates from normal cells of the body or food uptake [3] and only a small fraction representing <1% of cfDNA is related to tumors. This so called ctDNA is delivered from primary tumors, metastatic sites, or CTCs. It should be noted however, that CTCs and ctDNA have overlapping roles in oncology such as diagnosing cancer, detecting progression, and predicting treatment response [4]. Comparison of the main features of CTC and ctDNA are presented in Table 1.

In several studies, single CTCs and ctDNA were concurrently isolated from patients with solid tumors to evaluate the genetic heterogeneity of tumor types. Compared to ctDNA, higher heterogeneity was found in CTCs; moreover, when the alterations present both in CTCs and ctDNA, these were sometimes undetected in the primary cancer. Blood-based tumor compartments showed a higher degree of concordance with the metastatic tumor than with the primary one. Since most cancer patients die due to metastases, it was not an unexpected finding that the alteration detected in CTCs or ctDNA correlated with worse survival. The majority of the observed DNA aberrations were detected consistently in the case of repeated liquid biopsy samples. In most cancers, CTCs and ctDNA were found to be able to predict therapeutic response, disease progression and overall survival (see Table 2) [8,9,10].

It is important to understand when to use ctDNA or CTC. The ctDNA test usually requires prior knowledge of the target of interest. Moreover, not all DNA mutations are expressed. As for CTC, the intact tumor cell may originate from a resistant clone; thus, its DNA could provide valuable information about the mechanism of resistance as well as support therapeutic decisions. Moreover, CTC can be multiplied in culture to evaluate drug resistance both in vitro and in vivo. Overall, CTCs may prove to perform better at identifying novel targets and frequency of multiple known targets, whereas targets identified by ctDNA may be useful in clinical trials (see Table 2). An important point is to consider is that isolation of CTC is more costly and technically more challenging than that of ctDNA.

Liquid biopsy samples are potentially suitable for molecular profiling. Circulating tumor cells correspond to the population of metastatic cells; however, it is still uncertain whether circulating cfDNAs accurately represent the primary cancer and/or metastases. Moreover, it remains unclear whether cfDNA fragments provide appropriate information to diagnose cancer or select treatments for a given patient. The aim of this review is to evaluate the present status of the use of cfDNA from these two points of view. Just in 2021, searching PubMed for the terms „liquid biopsy cancer”, “cfDNA”, and “ctDNA”, 1690, 1790, and 1500 publications were found, respectively. There is likely to be some overlap between them, but the sheer number of publications highlights the increasing importance of liquid biopsy in cancer research. In this article, our aim is to summarize the potential of these techniques for decision making in medical oncology targeting clinical oncologists primarily. It is not our goal to give detailed technical information concerning preparation of cf/ctDNA, but instead we want to improve the understanding of the clinical oncologists who are not experts in molecular biology, genetics, and next-generation sequencing (NGS). Regarding the technological advances in the methodology including analytical aspects and research areas, we refer to recent comprehensive reviews [10,11,12,13,14,15,16].

Since molecular biology data is integrated more and more in the decision making of precision oncology, the clinical oncologist should gain insight into the many potential applications of cf/ctDNA. It is important to note, that the maturation of liquid biopsy will require multidisciplinary cooperation. The clinician should work together with the molecular biologist, geneticist, and bioinformatic scientist in routine oncology care [17].

**Table 2:** Future possibilities in the clinical management of ctDNA in solid tumors [18].

Tumor Type	Screening	Early Stage	Monitoring for MRD	Metastatic Disease	To Prove Therapeutic Effect	Targeted Therapy Indicated Based on a ctDNA Test
Bladder [19,20,21]	Liquid biopsy in muscle-invasive bladder cancer (MIBC) and nonMIBC urinary-cfDNA for those, who are not taking flexible cystoscopy [22]	Yes	Yes	Yes	Yes	Ongoing trials [23]: deraazantinib, erdafitinib, futibatinib, infigratinib, lenvatinib, pemigatinib, regorafenib, regoratinib,
Breast [24,25,26,27,28]	ctDNA assays hold substantial potential as an early cancer screening test, but very early stage (asymptomatic) tumors are not likely to release enough ctDNA to be detectable in a typical blood draw of 10 mL [29]	No	Yes	Yes. Brain and meningeal metastasis from cerebrospinalfluid ctDNAs [30,31]	Yes	Alpelisib [32]; Ribociklib, Neratinib, abemaciklib Trials ongoing: PADA-1, SOLAR-1, MONALEESA-2,3,7; BELLE-2,3; PALOMA-3, POSEIDON, SUMMIT, BEECH, I-SPY2, MONARCH2, LOTUS, INSPIRE, Neo-ALTO, MONAL
CRC [33,34,35,36,37,38]	Yes	Yes	Yes	Visceral metastases were found to be associated with detectable ctDNA [39]	Yes	Trials ongoing [40]: CIRCULATE-Idea; GALAXY, ALTAIR, DYNAMIC, TRACC, MEDOCC-CrEATENRG-GI-005, VEGA, ACT-3, IMPROVE-IT2
Esophagus [41,42,43]	Not good for screening [34]	No	Yes	Yes	No [44]	Afatinib, crizotinib, ABBV-321 trial: serclutamabtalirine [45]
Gastric cc [46,47]	Yes	Yes	Yes	Yes	Yes	–
Head and neck [48,49]	Not good for screening	No	Yes	Yes	After radiation therapy [50]	–
Liver/bileduct [33,51,52,53]	Yes Even from the bile fluid [54]	Yes	Yes	Yes	Yes	Trials ongoing in HCC [55]: Sorafenib, sunitinib, cedirabinib, linifanib, dovotinib, brivanib
Melanoma malignum [56,57]	No data	No data	Yes	Yes	Yes	Dabrafenib plus trametinib [58]
NSCLC [59,60,61,62,63]	Yes	Yes	Yes	Yes	Yes	Durvalumab, amivantamab [64]
Ovary [35,65,66]	Specificity and sensitivity is better than if CA-125and HE combined wihcfDNA [67]	Not yet	Not yet	Yes	Yes	–
Pancreas [68,69,70]	Not good for screening [71,72]	No	Yes	Yes	ctDNA and CA19- showed similar trends	–
Prostate cancer [73,74]	Yes	Yes	Yes	Yes	Yes [75]	–
SCLC [76,77]	Yes	Yes	Yes	May predict the progression of lung cancer patients earlier than imaging [78]	yes	–

## 2. Liquid Biopsy

The term of liquid biopsy refers to obtaining any tumor-derived material from blood or other fluids (e.g., such as cerebrospinal fluid, pleural effusion, ascites, saliva, bile, stool, and urine) [79,80]. Liquid biopsy is a minimally invasive method to yield cell-free RNA and DNA, tumor-related exosomes, extracellular-membrane-encased vesicles, and circulating tumor cells for further analyses. It is important to note that cell-free RNA is less stable than DNA [81]. Another possible definition could be that liquid biopsy is the analysis of biomarkers in non-solid biological tissue, where cfDNA as a term refers to fragmented DNA obtained from blood as a noncellular component.

## 3. Cell-Free DNA (cfDNA)

The cfDNA in healthy persons is mainly found as double-stranded fragments of approximately 150 to 200 base pairs in length, as fetal and maternal samples show [82]. The cfDNA was first described in 1948 [83], whereas elevated concentrations of disease-related cfDNA were reported in systemic lupus erythematosus in 1966 [84]. The relationship of cfDNA with cancer was reported in 1977 [85]. Somatic point mutations in cfDNA were identified only in 1994 [86].

The cfDNA fragments are thought to originate mostly from apoptosis and necrosis, both of which can occur in cancer tissue. There are three categories and three sources of circulating DNA in colorectal cancer, normal extratumoral cells, tumor microenvironment cells, neoplastic cells, and necrosis, apoptosis, and active secretion, respectively [87].

cfDNA levels can increase due to tissue injury caused by surgery, inflammation, or even strenuous exercise [88]. Other factors influencing cfDNA levels might include ethnicity, gender, age, smoking, body-mass index, and diet [89].

Moreover, cfDNA fragments of nontumor origin may increase during sample taking, sample processing, ordue to hemolysis [90]. A recent study investigated the day-to-day and within-day biological variations of cfDNA in healthy volunteers (n = 33) as well as in cancer patients (n = 10) [91]. Plasma samples were taken over three days and on the second day, every third hour for 12 h (noon, 3 pm, 6 pm, 9 pm). The between- and within-subject variation was close to each other, 30% and 25%, respectively. No systemic difference from day-to-day levels was observed regarding cfDNA (*p* = 0.061), but a significant decline was detected during the day (*p* = 0.03).

The plasma half-life of these cfDNAs is possibly dependent on many processes (e.g., plasma nuclease activity, physiological or pathological conditions such as pregnancy, hemodialysis, cancer); however, the exact method of the elimination is still not clearly known. It is believed that urinary excretion could play an important role [92]. The cfDNA can be used in cancer because the available data suggest that in the case of cancer, the amount in plasma is elevated compared to healthy individuals. In healthy people, the average cfDNA levels in plasma are found to be in the range from 0.3 to 15 ng per milliliter and cancer patients have increased levels compared to persons without cancer [18,93]. The timing of taking the blood sample might influence the variability of cfDNA yield; however, this study investigated only sixteen healthy volunteers [94]. Blood samples were taken three times (7 am, noon, 5 pm), and for most subjects, maximal values of cfDNA were observed at midday, but maximum values also occurred in the morning or afternoon, revealing no statistically significant differences between the timepoints. The clinical course might also influence the timing of liquid biopsy, e.g., sample taking at the time when the cancer is responding to therapy might reduce the sensitivity of the ctDNA test.

Circulating tumor cells are also sources of cfDNA, but the amount produced by this route is variable: from less than 0.1% to more than 90% of the total amount of cfDNA [95]. In prostate cancer, it was shown that there is a relationship between circulating tumor-associated DNA in the blood and the occurrence of CTC [96]. On the other hand, it seems that cfDNA significantly decreases in the blood following anticancer treatments such as surgery, surgery and radiation, neoadjuvant chemotherapy, and surgery by the end of all these treatments [97].

Due to the short half-life and low level of cfDNA, special ultrasensitive techniques were developed for isolation as well as analysis, but a detailed description of these are out of the scope of this paper; thus, we are referring to some review papers [98,99,100,101].

The cfDNA pool also contains mitochondrial DNA released by the tumor [102,103,104]. Circular double-stranded mtDNA exists in the energy producing center of mitochondria up to thousands of copies and contains a significant amount of unmethylated DNA. This cell-free mitochondrial DNA (cfmtDNA) is still not well characterized; however, the cfmtDNA seems to be more fragmented and its size ranges between 40–300 bp [105]. There are data, however, about cfmtDNA in both healthy volunteers and cancer patients [106,107]. Based on the mitochondrial DNA, it may be possible to differentiate cancer patients from the controls [108].

It is of interest, however, that the amount of cfDNA decreases by approximately 90% following radiation therapy indicating that necrosis might not be the primary pathway of cfDNA release, since after radiation, an increase would be expected [18]. A potential explanation of this phenomenon is the observed reduction due to radiation-induced inhibition of cfDNA release pathways in healthy cells [109].

Apart from cellular destruction, some studies indicate that cfDNA may be also derived from active cellular secretion [110,111]. Data obtained from in vitro cell culture studies have shown that cfDNA can be found in the culture medium. Its level did not correlate either with cellular destruction (apoptosis, necrosis), or with DNA replication [112,113]. The secreted cfDNA fragments are usually in the range of 1000–3000 bp which is different from those associated with apoptosis or necrosis [114]. The exact mechanisms of this active release of cfDNA are still obscure but it might be due to genomic instability [115].

There is another regulated source of DNA release into the human blood, namely fragments associated with extracellular vesicles, such as exosomes. These vesicles carry cfDNA fragments ranging from 30 bp to 20,000 bp [116,117,118]. The problem with evaluation of exosomal DNA is that the ratio of cfDNA localized in the interior of vesicles vs. the ones bound to the exterior surface is not yet exactly determined. This makes it difficult to analyze the cfDNA content of exosomes playing a role in cell regulation.

It is now clear that cfDNA originates from many sources and is released to the blood through various mechanisms. These mechanisms could be modulated by several environmental and/or biological factors; thus, cfDNA may be unique to each individual. Consequently, individual monitoring of cfDNA might offer the best information from a given patient. It has clinical relevance that the application of cfDNA testing is growing rapidly, but no practice guidelines exist [119].

Due to the lysis of the normal cells in the sample, contamination continuously can occur, since the isolated cfDNA fragments from the plasma will contain fragments of the normal cell DNA. In the isolation of ctDNA from cfDNA, the two critical issues to be considered are stability and purity. Consequently, quick removal of the cells, as well as obtaining the cfDNA fraction from the plasma is important. In fact, the purity and cfDNA concentration shows correlation with processing time and may increase the variability of the results; thus, it makes the interpretation of data difficult [120]. To overcome this problem, cfDNA collection tubes were developed which contain additives stabilizing both cfDNA and the normal cells for up to 14 days at room temperature [121]. In the case of cancer patients, the ratio of cfDNA originating from normal and cancer cells is very variable but generally the amount of ctDNA is low, namely, among all alleles (including wild-type alleles), the percentage of variant alleles is scarce [122]. In fact, the ctDNA is extremely under-represented compared to the massive background of normal cfDNA, and thus limited in yield. However, the modern techniques are able to identify and quantify alterations of allele frequencies in cfDNA of 0.01% or less [123]. In healthy individuals, it is now possible to detect genomic alterations from cfDNA with a limit as low as 0.08% allelic frequency; thus, screening for cancer by cfDNA is possible [124]. It is important to note that different cancers could share common mutations (e.g., KRAS, BRAF, TP53); thus, detecting these from ctDNA will not give information from the location of the primary tumor [125]. Moreover, cfDNA from benign lesions may also contain mutations found in cancers that will make the proper evaluation of the cfDNA test result harder. In fact, it was shown that benign nevi contained the same BRAF mutation found in melanoma [126]. The detectable mutations in cfDNA are often derived from either aberrant or benign clonal populations of the bone marrow [127]. The frequency of clonal hematopoiesis of indeterminate potential (CHIP), as this phenomenon is termed, could increase significantly with age, and over the age of 70, may occur in more than 10% of patients [128,129]. There are data indicating that parallel sequencing of white blood cells might overcome the problem of mutations originating from CHIP. It is important to understand that variants detected in ctDNA test could be either of germline origin or may not be related to tumor, e.g., CHIP. Follow-up testing of leukocyte DNA could decide if the observed variant is associated with CHIP. This is important since CHIP is a frequent cause of false-positive results in ctDNA tests. Finally, resistant clones to therapy frequently develop in cancer tissues and may overgrow the sensitive cells. In fact, in a retrospective analysis of cfDNA from 21,807 late-stage cancer patients who received therapy and represented more than fifty cancer types revealed sub-clonal structures as well as emerging resistance [130].

The acquired resistance is frequently characterized by outgrowth of multiple resistant subclones in a patient receiving therapy [131]. These resistant clones can occur in the primary tumor or in a distinct metastatic site. Consequently, tumor biopsy of a single lesion might under-represent the tumor heterogeneity while the cfDNA, originating from all tumor tissues, reflects it more accurately. In fact, data available comparing multiple tumor biopsies with cfDNA obtained from a single plasma sample can cover all unique alterations observed in different metastatic sites [132].

In a study, data was obtained by both tissue sampling and liquid biopsy of 229 lung cancer patients [133]. The liquid biopsy detected 82 (35.8%) while the tissue biopsy identified 47 (20.5%) patients with targetable mutations. Thus, liquid biopsy using cfDNA/ctDNA might be a suitable alternative to tissue biopsy for those who have no access to tissue biopsy (e.g., high risk of tissue sampling) or the tissue biopsy sample is of low quality. Using repeated liquid biopsy for monitoring the patient’s disease state may also lead to early detection of emergent genetic alterations driving acquired resistance to the applied therapy and a provide foundation to therapy switch. In a study, early signs of secondary drug resistance were detected by monitoring cancer patients over a two-year period [134].

Considering that liquid biopsy is a minimally invasive intervention and easily repeatable, it might be used for longitudinal studies monitoring progression from pre-cancerous states to transforming to cancer.

In a recent paper, 1370 cancer clinical trials were identified which involved liquid biopsy. The data obtained regarding early detection is promising, by observing progression, or real-time monitoring of the development of acquired resistance by analyzing cfDNA [135]. There are, however, several issues to standardize before applying liquid biopsy in clinical oncology as a routine test including blood collection tubes, handling the obtained sample material, isolation, and quantification of cfDNA.

Due to the accumulation of data on DNA methylation, a new paradigm emerged regarding not only cellular regulation but also for the treatment of diseases. DNA methylation is an epigenetic modification which is necessary to the normal genome regulation and development. In fact, the role of epigenetics is to study those heritable changes that occur in the phenotype or gene expression but not due to the changes in the primary DNA sequence. There are other recognized epigenetic mechanisms as well including microRNAs, long noncoding RNAs, chromatin remodeling, and post-translational modification of histones [136,137,138]. These epigenetic mechanisms contribute to the regulation of several important molecular processes in the nucleus including repair, replication, RNA processing, and transcription, as well as modulating the chromatin structure.

The vertebrae genomes are predominantly methylated at the dinucleotideCpG. The CpG sites are those regions of DNA where a cytosine (C) nucleotide is followed by a guanine (G) nucleotide in the linear sequence of bases, in 5′ → 3′ direction and not a C–G bound between the two DNA strands. When the CpG sites occur with high frequency in certain parts of the genome, they are called CpG islands (or CG islands). In the CpG, the C can be methylated by adding a methyl group to the fifth carbon of C (5mC) via DNA methyltransferase. In fact, in mammals the C-s are methylated at the CpG sites 70–80% [139]. Similar data was obtained regarding normal human cells. In the human fibroblast cell line, the C is methylated 4.25% in the whole genome, the CpGs were methylated 67.7%,and 99.98% of mC occurred in CpG. In human embryonic stem cell lines, these were 5.83%, 82.7%, and 25%, respectively [140].

The methylated-C is further modified by the TET (ten-eleven translocation) enzymes. In this process, 5mC is oxidized to 5-hydroxymethylcytosine (5hmC), 5-formylcytosine (5fC), and 5-carboxylcytosine (5caC). The regeneration of 5fC and 5caC to cytosine is possible due to the base excision repair as well as the thymidine DNA glycosylase pathway. It is important to note, that methylating the C can change the expression of a gene, and the methylated-Cs often mutate to thymines; both of these mechanisms can contribute to the development of cancer. In normal health status, the CpG islands are mostly unmethylated, but the CpG dinucleotides outside CpG islands are usually methylated. In the case of malignant transformation of the cells, these methylation patterns reverse to the opposite, namely, the CpG dinucleotides become hypomethylated outside the CpG islands and hypermethylation of CpG islands occurs [141]. Moreover, in humans about 70% of promoters can be found near to the transcription start-site of a gene (proximal promoters) and contain CpG islands [142,143].

It is known that at the genomic level, the onset or the progression of a tumor causes changes in DNA methylation that proves that epigenetic modulation occurs in cancer. The importance of epigenetics in cancer is underlined by the fact that in certain epigenomic environments, the loss of p53 function or the tumor-driving effects of KRAS could be promoted [144,145]. The aberrant methylation of DNA is an early event of tumor development, and it is present abundantly during the entire disease process [146]. In fact, the cancer-specific changes of DNA methylation could occur even before the occurrence of gene mutations during tumorgenesis [147]. The main difference between normal tissue and cancer is that cancer tissues contain much less hydroxymethylC (hmC). In fact, almost any type of cancer tissue shows a highly significant reduction regarding hmC, but there is only a mild loss of mC [148]. It is still not known why hmC is lost in the case of cancer. One potential explanation is that due to the fast proliferation the enzymatic machinery (TET proteins) may be exhausted. In contrast, it is known that dormant cancer cells are also negative for hmC. This might help to detect non-proliferating cancer cells. One of the main aims of liquid biopsies is to detect cancer even if the imaging tests are negative. It is important to note, that the methylation patterns are usually tissue-specific; thus, the distinct hmC patterns make it possible not only to identify cancer but also the tissue of origin, which is important in the case of unknown primary tumors. Finally, the characteristic cancer mutations may occur in a relatively low number of genetic locations, but there are about 30 million methylation sites scattered around across the human genome; thus, they provide a rich signal for cancer detection [149]. Unfortunately, there are limitations to the cfDNA analysis, since the epigenetic alterations found in cancer may also occur in noncancer tissues, resulting in false positivity [150]. As an example, several methylation alterations are shared by esophageal cancer and Barrett’s esophagus [151]. False-negative outcomes are also possible if the detection signaling is below the limit of detection. The use of the methylation pattern of ctDNA, however, could acquire an important role in oncology. In a recent paper, it was shown that based on ctDNA methylation profile discrimination among intracranial tumors (e.g., IDH mutant gliomas, IDH wild-type gliomas, meningiomas, hemangiopericytomas, low-grade glial-neuronal tumors, and brain metastasis of unknown primary cancer) is possible [152]. This is important since invasive neurosurgical intervention for diagnoses might be avoided by this approach.

It should be noted, however, that cfDNA may contain exceedingly low allele frequencies which are close or below the error rate of the applied techniques (PCR, NGS). The molecular barcoding techniques may overcome this problem [153]. Moreover, optimization of this method might lead to the detection and analysis of ctDNA in the clinical practice. This is because ctDNA analyses can provide much more information than just mutation analysis including size fragment patterns, transcriptomics, methylation status, or even viral load [154].

## 4. The Circulating Tumor DNA (ctDNA)

Personalized therapy in oncology based on the knowledge that the molecular changes in the background of a patient’s tumor provide a chance to selectively target these genetic changes in the cancer cells with curative intent. Thus, targeting cancer-related mutations becomes a fundamental part of oncology [155]. The ctDNA together with tissue samples or CTCs are the source to find out these cancer-related genetic alterations.

The amount of ctDNA reflects tumor burden, namely, sums up to 1% in early-stage cancer and up to 40% in late-stage disease. It is important to note, that the amount of tumor burden and the level of ctDNA do not necessarily reflect each other, indicating differences in the rate of cell death in different types of cancers [156]. Thus, ctDNA could only be detected in individuals having cancer. Moreover, the ctDNA fragments obtained by liquid biopsy contain epigenetic abnormalities, e.g., methylation changes, as well as genetic alterations characteristic to the primary cancer which can be utilized in diagnosing the type of the tumor, selecting appropriate treatment, detecting recurrence, or predicting the prognosis [12]. Thus, ctDNA could be applied for different objectives at different timepoints during the course ofthe cancer. There are attempts for the monitoring as well as adapting of cancer treatment by using ctDNA kinetics [157]. To achieve clinically useful ctDNA kinetic tools, however, requires validated measurement methods, timepoints, and advanced bioinformatics.

Moreover, it seems that the ctDNA (~134–144 bp) that were reported are shorter than that of cfDNA (~166 bp) [157,158]. Others found the opposite, namely, the cfDNA of apoptotic origin consist of fragments shorter than 1000 bp, while fragments released by exosomes, or necrotic (tumor) cells are longer in size over 1000 bp [109]. In fact, data indicate that the vast majority of ctDNA in plasma originates from apoptosis. Consequently, their size is in the range to be nucleosome protected DNA (range 120–220 bp; peak around 167 bp) [159]. It should be kept in mind, however, that the ctDNA could contain very long-sized fragments (~10,000 bp) due to the necrosis of tumor cells [160]. If larger DNA fragments are increased in plasma, that might result in false negativity due to interference with the detection of ctDNA. 

Since the discrimination of ctDNA from the normal cfDNA based on mutation hotspots is limited, it seems that the analysis of the methylation of ctDNA is a more sensitive approach for diagnosing cancer as well as predicting prognosis [161,162]. DNA methylation is a covalent modification that changes gene expression consequently. DNA methylation is a mechanism for transmitting and perpetuating epigenetic information through DNA replication and maintaining that during cell divisions. Thus, it became a therapeutic target in cancer and other diseases [163,164]. The methylation patterns of DNA are stable and do not change in purified genomic material. The DNA or cfDNA, and ctDNA methylation pattern is thought to be a sensitive and reliable method not only for the diagnosis of cancer but it may also be a prognostic marker [165]. Recently, several technologies were developed to study the methylation of cell-free DNA (methylome) including next-generation sequencing, genome-wide methylation profiling, and DNA methylation analysis [166]. Since during bisulfite DNA sequencing, false positivity is a concern, it is rarely used today. The new technologies do not need bisulfite treatment of the DNA. NGS is suitable for genome-wide methylation studies because DNA methylation sites in a single-base resolution could be detected [167]. The cfDNA methylation analysis in cancer has the potential of clinical applications, e.g., investigating single-gene methylation profiles in different cancer types [168]. Recently emerged the possibility to develop a single test for early cancer detection (stMCED) by using ctDNA methylation fingerprints as a biomarker of malignancies [169]. The “methylome” leads to a better understanding of the onset and the phenotype evolution of a cancer. Recently, it was shown that tissue damage caused by metastasis induces the release of cfDNA from affected tissues to the circulation and this can be detected by using tissue-specific methylation markers [170]. This is a breakthrough, if confirmed, because then liquid biopsy is not only able to detect metastatic disease but also to suggest the tissue location of metastases. This method may be used to find the location of a cancer of unknown primary origin [171].

## 5. The Applications of cfDNA and ctDNA in Clinical Oncology

It would be important if ctDNA could replace tumor tissue biopsy since obtaining fluids is easier, less risky, and less painful. Thus, liquid biopsy makes it possible to monitor the tumor DNA over time and makes it also possible to alter the treatment if necessary. This is especially important if the patient has several metastatic sites because ctDNA may represent the heterogeneity of metastatic sites better than tissue biopsy from one metastasis. In fact, liquid biopsy could represent the whole picture of a metastatic or advanced cancer patient’s malignancy [172,173]. Consequently, selecting treatment for multiple metastatic patients might lead to better outcomes if the decision making is based on the results of liquid biopsy, but this is still to be proven. In fact, in some studies, low concordance regarding DNA alteration comparing tumor and plasma samples from the same patient are suggested [174,175]. Large-scale, well-designed studies show an 80–90% concordance between DNA samples obtained from plasma and tumor simultaneously [176,177]. Moreover, when the mutation frequencies of cfDNA and tissue sequencing databases were compared in colorectal cancer, it was found to be closely matched [178]. The same study also revealed that regarding anti-EGFR mAb therapy, patients may harbor up to thirteen different resistance alterations and less than 10% of colorectal cancer patients have only one resistance alteration. Thus, the cfDNA and ctDNA test might become a valuable tool for detecting resistance to a drug before initiating or deciding to rechallenge with anti-EGFRtherapy [179]. The driver genes are expected to be more important in time regarding the decision making in oncology [180].

However, available data shows that in the case of metastatic cancer, approximately 15% of the samples taken will not contain enough cfDNA for molecular profiling [181,182]. If there is enough plasma DNA in the biopsy, the level seems to correlate over time with either the tumor burden or response to therapy. In a prospective study, the results of tumor biopsy and liquid biopsy were compared in 42 gastrointestinal cancer patients following progression, regarding possible acquired resistance alterations. Investigating cfDNA, the data revealed that in the case of liquid biopsy, when clinically relevant resistance alterations or even multiple resistance mechanisms were found in the matched tumor biopsy, then no resistance was detected in 78% of cases [183].

In early stage TNBC (triple-negative breast cancer) revealed that the probability of distant disease-free survival at 24 months for ctDNA-negative and positive patients were 56% and 81%, respectively [184]. The follow-up of TNBC patients who received neoadjuvant therapy showed that the first sign of metastatic disease—irrespective of other signs or symptoms—in about three quarters of patients, was the persistence or reappearance of ctDNA [185].

These data obviously predict the increasing role of liquid biopsy in the near future.

**Figure 1:**
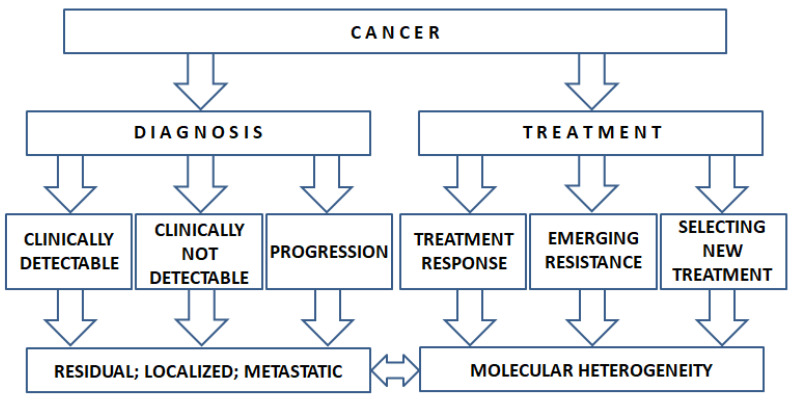
The potential use of ctDNA in clinical decision making.

In the era of targeted therapy, the molecular profiling of cancer DNA has become a standard approach in oncology. The use of ctDNA could increase the number of patients receiving targeted therapy. The ctDNA test could help in selecting the most appropriate treatment considering not only the efficacy but also the issue of potential drug resistance [181]. Due to the low risk of obtaining ctDNA, the test can be useful in several clinical scenarios (Figure 1).

Data are accumulating that plasma-derived ctDNA contains genetic material which is representative of the tumor genetics. In fact, the mutations observed in the primary tumor as well as in the metastatic sites can be captured by ctDNA. Thus, ctDNA is suitable not only for diagnosis but also for selecting an appropriate targeted therapy for the individual patients [116]. It is important to note, however, that among cancer patients with the same type of malignant disease, there is significant variability in the amount of ctDNA reflecting the biological differences of the dynamics of cell death in the individual tumors. Moreover, in the case of different tumor types, the frequency of detecting ctDNA shows great variation. 

## 6. Cell-Free DNA in Diagnosis

The half-life of cfDNA is short; consequently, it may be appropriate for monitoring the current tumor burden in response to therapy. In fact, the plasma half-life of the circulating cfDNA including ctDNA is between 16 min and 2.5 h. Thus, liquid biopsies seem to allow real-time monitoring of the tumor burden [186]. This would be a great advantage since the serum half-life of standard tumor markers such as CA-125 and CEA is days or weeks [37]. There are data indicating that changes in ctDNA may be more accurate in the prediction of treatment response than traditional tumor markers [187].

The mechanism of clearance is poorly understood but it may involve DNase activity, uptake by the liver and spleen following macrophagic degradation, or renal excretion [188,189,190,191]. The clearance could be further influenced by the association of the cfDNA fragments to serum proteins including C-reactive protein, albumin, HDL, transferrin, prothrombin, fibrinogen, fibrin, etc. [192]. The clearance might be further altered by the fact that cfDNA can be recognized by different cell surface DNA-binding proteins following celluptake for possible degradation [193].

Thus, a machine learning model was developed (DNA evaluation of fragments for early interception—DELFI) for detecting ctDNA by genome-wide analysis of cfDNA fragmentation. In this prospective study, the fragmentation profile of 236 cancer patients having different tumors (breast, colorectal, lung, ovarian, pancreatic, gastric, or bile duct) were compared to data obtained from 245 healthy individuals. The sensitivity of this model was different among cancer types (ranging 57–99%) at 98% specificity. In 75% of patients, the fragmentation profile was useful to detect the tissue of origin. By combining DELFI with mutation-based cfDNA, the cancer detection rate was further improved (91%) [194]. It is believed that the sensitivity of ctDNA tests could be enhanced if combined with methylation or fragmentation patterns [195,196].

In a recent study, the same model was applied to study the fragmentome of lung cancer [197]. During this prospective study, individuals who had a risk for lung cancer (heavy smokers, age 50–80 years) participated. The fragmentome was analyzed together with CEA level, clinical risk factors, as well as CT imaging was carried out. Out of 365 individuals studied, 129 were found to have lung cancer. The DELFI score of the cancer patients was not affected by acute or chronic inflammation. Moreover, despite of the small number of SCLC, data indicates that the analysis of the fragmentation profile may be able to differentiate among lung cancers of different histology. Additionally, the ctDNA detection could be a powerful biomarker for observing minimal residual disease or relapse of NSCLC. Applying post-treatment surveillance, the sensitivity and specificity to detect relapse ranges from 82–100% and 70–100%, respectively [198].

A recent meta-analysis on 27 studies including 3459 patients showed that post-operative ctDNA is a strong prognostic marker of RFS in CRC [199]. The ctDNA serial monitoring of patients with negative radiographic evidence of the disease might outperform the traditional pathologic prognostic approach for the evaluation of risk of recurrence in CRC. The ctDNA test also allows for the early detection of relapse in stage II colon cancer [200]. The ctDNA testing might also be useful to detect MRD across luminal GI malignancies, in particular CRC [201].

Breast cancer diagnosis is based upon immunohistochemistry. Data indicate that following neoadjuvant treatment, the ctDNA clearance was associated with a higher rate of complete pathological response. The ctDNA test is able to detect early breast cancer [25]. In metastatic breast cancer, ctDNA can guide the optimal treatment sequence to be followed [24]. In breast cancer, the cerebrospinal fluid is used to detect leptomeningeal metastasis; however, this may require repeated lumbar puncture. Thus, plasma ctDNA analysis of 30 patients with known leptomeningeal metastasis was carried out to assess the potential of ctDNA in diagnosing this disease stage. Plasma ctDNA yield was limited to patients who had previous whole-brain irradiation and had extracranial disease progression [28].

Among gynecological cancers, the endometrial cancer is the most frequently occurring and ovarian cancer is the most lethal one. At present, the final diagnosis of these tumors is based upon histopathology obtained from the tumor tissue. Recently, the use of cfDNA in both ovarian cancer (OC) and endometrial cancer (EC) was explored [202,203]. These papers revealed that the use of cfDNA in OC is limited to the advanced stage or types of OC, and the ctDNA in EC patients was detected in only 42.2% of cases mainly with aggressive disease. Thus, further large-scale studies are needed to evaluate the applicability of cfDNA in these indications especially in diagnosing early-stage cancers.

Despite prostate cancer (PC) being the primary cause of death among men in developed countries, screening by prostate-specific antigen has a low specificity (about 15% of asymptomatic PC patients do not present with elevated PSA). Thus, more sensitive biomarkers are needed. Methylation of the CpG islands occurs in PC [204]. In fact, DNA aberrations found on the androgen receptor gene detected by cfDNA strongly correlated with the outcome of castrate-resistant PC patients [205]. Targeted sequencing using CTC or cfDNA is applied to guide androgen-directed therapy [206]. The amount of cfDNA did not relate to the presence of PC, but higher amountswere found in advanced disease [73]. The same study, however, revealed that there are some potential markers to identify aggressive forms (HOXD8rc, CXCL14, SLC16A5rc, and GRASP) or progression (DOCKK2, HAPLN3, and FBXO30) of PC, but the methylation of some of these markers found in the tissue samples was not detected in ctDNA. These data indicate the uncertainty of using ctDNA in PC. In a study, target sequencing of 182 serial ctDNA samples from 53 advanced urothelial cancer patients was performed [207]. The serial ctDNA data and monitoring the variant allele frequencies was combined with clinical factors. An increase of ctDNA aggregate variant allele frequencies by ≥1 predicted disease progression within five months in 90% of patients. However, patients with ctDNA aggregate variant allele frequencies ≤0.7 achieved more than six months of clinical responses. Variant allele frequency (VAF) of gene mutations is defined as the number of variant reads divided by the number of total reads, reported as a percentage. The information on VAF is thought to be important since it may provide evidence of the subclonality of a variant. The subclonal variants are believed to insufficiently react to a therapy targeting the variant. A recent meta-analysis indicates that a high VAF is an independent, adverse prognostic factor for OS in TP53-mutant MDS patients [208]. If the ctDNA is low in the sample, that limits the reliable assessment of VAF. At present, VAF is not to be used in clinical decision making because of a lack of clear evidence that true subclonal variants are predictive of lack of response.

One of the most important aims of using ctDNA in clinical oncology is to detect the presence of a tumor without clinically evident disease. Data indicate that the level of circulating tumor DNA may be increasing weeks or months before progression detected by imaging services [209]. If tumor-specific mutations persist in cfDNA samples taken four weeks following surgery that could be considered as evidence of residual tumor. The relationship between post-operative detection of tumor-specific mutations in cfDNA analysis and residual disease, as well as tumor relapse, was also proven in the case of breast, lungand pancreatic cancer [25,78,210]. In advanced esophageal cancer, ctDNA is highly diagnostic but it is not appropriate for early diagnosis of esophageal cancer [41]. Among colon cancer patients with stage II disease who did not receive adjuvant chemotherapy, those who had persistent tumor-specific mutations detected in the liquid biopsy had a risk of residual tumor which was 18 times higher (*p* < 0.001) compared to patients with undetectable tumor-specific mutations [201]. In stage III colorectal cancer, using serial ctDNA measurements could be used for risk stratification since repeated ctDNA measurements showed a strong correlation with tumor growth rate [211]. These data indicate that ctDNA may be useful to detect clinically silent tumors of individuals thought to be healthy. In a systematic review analyzing the literature, it was found that ctDNA could be useful for predicting the treatment response of neoadjuvant chemo-radiotherapy, as well as potentially serving as a prognostic marker for locally advanced rectal cancer [212]. Moreover, ctDNA analysis may play an important role in clinical trials influencing trial designs. In fact, in oncology clinical trials, the concept of ctDNA-based molecular residual disease is introduced [213]. Data is accumulating that in the case of adjuvant immunotherapy, the benefit is restricted to ctDNA-positive cases [214].

Unfortunately, liquid biopsy for the detection of early stages of tumors is still a problem sometimes due to the small tumor bulk since the low plasma ctDNA may be undetectable with the methods available today [215].

## 7. The cfDNA and ctDNA in Cancer Treatment 

There is scarce data available that the ctDNA level could increase transiently following targeted therapy and this may indicate the effectiveness of therapy [216]. Quick temporary increases of ctDNA level might also occur following chemotherapy indicating increased release but not necessarily indicating the therapeutic outcome. Moreover, if the therapy is effective, the cfDNA level decreases significantly within 1–2 weeks. Early decrease of ctDNA after initiation of therapy might not be the sign of effective therapy but the temporary inhibition of ctDNA release. Therefore, the repetition of the ctDNA test at later time is suggested.

Detecting treatment response early may reduce the duration of time of insufficient therapy since the imaging control to evaluate treatment response generally occurs every 2–3 months. Regarding targeted therapy, it was found that resistance to the drug can be detected much earlier in cfDNA samples than observing by imaging or applying the standard tumormarkers [217,218]. Moreover, liquid biopsies seem to have the potential to discover novel cancer biomarkers for tumor diagnosis and prognosis [219,220]. The cfDNA test in ER-positive breast cancer revealed one or more ESR1 mutations indicating a poor outcome for an additional line of anti-hormonal treatment [221]. In advanced breast cancer, ctDNA analysis detected uncommon but targetable mutations of HER2 and AKT1 mutations; the first one being sensitive to neratinib and the second one being sensitive to capivasertib, indicating the clinical value of ctDNA tests [222]. In fact, in a recent clinical trial in metastatic triple-negative breast cancer, it was found that liquid biopsy is a quicker approach for molecular testing than tissue biopsy [223]. This is another advantage of liquid biopsy since clinical oncology is an area racing with time; the more quickly the adequate therapy is initiated, the better the outcome can be. In a study, the use of ctDNA was tested to predict response to neoadjuvant therapy in breast cancer. Blood samples were obtained prior therapy, following four cycles of chemotherapy or 12 weeks of aromatase inhibitor treatment (middle of the therapy (MT)) and at surgery (end of treatment (ET)). Prior to therapy, ctDNA was detected in 63/145 (43.4%) of patients. In the case of 25/63 (39.7%) patients, the ctDNA persisted at MT and 15/53 (23.8%) at ET. Among those with persisting ctDNA, there was significantly more residual disease detected at surgery. Moreover, out of 31 patients with detectable ctDNA during the treatment (MT), 30 patients were non-responders (96.8%). Thus, the authors concluded that persistent ctDNA during treatment may negatively predict the response in the case of neoadjuvant treatment of breast cancer [224]. A systematic review and meta-analysis investigating a similar question found that the detection of ctDNA at either baseline treatment or after completing neoadjuvant treatment is associated with significantly worse overall survival, HR 19.1, 95% CI: 6.9–53.04 and HR 4.00, 95% CI: 1.90–8.42, respectively [225]. An interesting finding of this paper was that the detection of ctDNA did not associate with the probability of achieving pathological complete remission. Thus, the use of ctDNA to evaluate neoadjuvant treatment is still obscure and requires further evaluation.

The potential of cfDNA in lung cancer was analyzed in 218 patients before the start of platinum-based chemotherapy and after two or three cycle of treatment [226]. Those patients who had the baseline value of cfDNA in the highest tertile had significantly worse disease-free (DFS) and overall survival (OS) rates compared to those with lower concentrations (median OS 10 months (95% CI, 10.7–13.9) versus 14.2 months (95% CI, 12.6–15.8), respectively; *p* = 0.001). Increased levels of cfDNA were found as an independent prognostic factor in multivariate analysis; however, the total cfDNA did not predict response to chemotherapy.

A good example for using ctDNA in therapy decision making is the identification of the emergence of the EGFR (epidermal growth factor receptor) T790M gatekeeper mutation (found in around 50% of lung cancer patients) by applying EGFR-inhibitory treatment in EGFR-mutated NSCLC (non-small-cell lung cancer) [227]. Another important finding of this study was that the cfDNA test could identify the coexistence of the T790M mutation and MET amplification, too, since these patients with the coexisting alteration might not benefit from changing to a third-generation EGFR inhibitor. It was shown also that the test results of tumor tissue tests and cfDNA of the same patients matched in high degree regarding the detection of the T790M mutation [228]. This single mutation can be successfully treated with third-generation EGFR inhibitors [229]. However, the clinical problem is that resistance mechanisms can also develop for the third-generation EGFR inhibitors which are different between patients and heterogeneous among tumor sites. NGS of cfDNA is suitable to clarify the resistance mechanisms of a given patient. To overcome this drug resistance, novel combinations [7,8] as well the fourth-generation EGFR-TKIs were developed [230]. In the case of SCLC, data indicate that genotyping by PCR could be inadequate since the EGFR L747_A755delinsSS exon 19 deletion was not detected by the real-time PCR but it was found by NGS [231]. This finding indicates the adequate comparisons of different methods used before implementing any of them in clinical practice. The programmed-death-ligand 1–programmed-death-1 (PD-L1–PD-1) inhibitors are proved to be effective for NSCLC patients with extensive PD-L1 expression or high tumor mutational burden. Thus, a new method was developed for analyzing the tumor mutation burden using ctDNA. The retrospective analysis of two large-scale clinical trials showed that those NSCLC patients can be identified who will benefit from atezolizumab therapy in the second line or higher [232]. In fact, recent studies in various cancer types such as colon cancer, melanoma, urothelial cancer, and NSCLC indicated that ctDNA analysis can identify those patients who can respond to immune checkpoint inhibitor therapy; moreover, the pseudoprogression under therapy may be separable from the real clinical progression [233,234]. The tumor mutational burden (TMB) is the total number of mutations found in the DNA of the cancer cells. It is considered as a special biomarker since it is believed that tumors with a high number of mutations may respond to certain types of immunotherapy. The data, however, are contradictory. In a recent paper, data of 10,000 patients with solid tumors were analyzed and did not support that high TMB is a biomarker for treatment with immune checkpoint blockade (ICB) in any solid cancer types [235]. Others, in smaller patient populations, found a correlation with high TMB and the favorable outcome with ICB treatment [236,237]. Data is accumulating that blood-based TMB, by analyzing ctDNA obtained by liquid biopsy, is feasible [238]. At present, immunotherapy should not be based on blood TMB alone. It was found that higher ctDNA TMB, at the current commercial sequencing length, reflects worse clinical outcomes in NSCLC [239]. Nowadays, in the case of metastatic NSCLC, ctDNA analysis could provide useful information in specific clinical scenarios [240]. The application of blood TMB is still uncertain and not a routine part of clinical decision making.

In the case of colorectal cancer, it was shown that patients with BRAFV600E-mutant cancer, the changes of this mutation level in cfDNA one month after the initiation of the targeted therapy shows a statistically significant correlation with response [241]. Namely, the responding patients showed a 90% or more reduction in ctDNA level. This is important since CEA did not show a correlation with treatment response. In fact, this data indicates that the cfDNA test is more suitable to predicting response to therapy than the standard tumor markers routinely applied in oncology. Moreover, liquid biopsy is a potentially useful technique to discover novel cancer biomarkers [221]. KRAS and NRAS mutations detected in metastatic colorectal cancer can predict negative response to anti-EGFR therapy. The ctDNA test could detect these mutations. Thus, comparison of two different methods of ctDNA mutational analysis (Beaming digital PCR [OncoBEAM] and IdyllactDNA qPCR) was carried out. In the study, 47 metastatic colorectal cancer patients participated who were previously tested for the RAS mutation using the tumor tissue. The overall agreement between the two PCR analyses was 91.7%. The concordance between tumor tissue and ctDNA analyses using the OncoBEAM and Idylla assays was 83% and 78.7%, respectively, which was improved to 96.2% and 88.5%, respectively, in treatment-naive patients. The authors concluded that analysis of ctDNA is a viable strategy for the clinical management of mCRC patients [242]. Data are accumulating that ctDNA in CRC patients might be used for surveillance since it detects recurrence 3–11.5 months earlier than imaging. The ctDNA decreases during first- or second-line therapy correlate with tumor response [37]. The ctDNA also has a high prognostic value in patients with resected CRC [243].

Detecting the high tumor burden is important from a clinical point of view since data are accumulating that this has a negative effect on cancer immunity. The ctDNA or circulating tumor cells bear the potential of indicating high tumor burden. Thus, measuring ctDNA might be an indicator for the use of immune-checkpoint inhibitors in the future [244].

The development of acquired resistance is one of the main obstacles of successful therapy in medical oncology. Acquired resistance is driven by the overgrowth of tumor cell clones with pre-existing resistance alterations [245]. Unfortunately, recent data indicate that many patients in fact, harbor more than one resistant subclone at the time of progression [27,246,247]. Since these resistant subclones may be found in the primary tumor or in the metastatic lesions [218]. Thus, the biopsy of one lesion may be unable to detect important resistance mechanisms developed outside of the biopsied region [187,248]. The ctDNA could be used to detect the emergence of acquired resistance [43,249].

## 8. Combined Analysis of ctDNA with Other Parameters

To expand possible applications of liquid biopsy, we must develop methodologies that enable simultaneous evaluation of ctDNA, ctRNA, CTC, and exosomes. In fact, the analysis of various samples obtained by liquid biopsy can be combined to improve the chances of more accurate information [250]. Scarce data are available regarding the combined use of the so-called circulome parameters. The circulome could reflect the tumor heterogeneity since these components (e.g., CTC, ECV, ctDNA, ctRNA) are derived from each cancer clone. Consequently, each blood sample reflects real-time information of the cancer [251]. The ctRNAs, EVs, and TEPs are relatively new players of the tumor circulome, but each has many promising potentials from diagnosis to prognosis and beyond at all stages of cancer [252]. The ctRNA could exist as free-floating, encased in exosomes, or carried by platelets [253]. Combined examination of RNAs contained in the exosomes and ctDNA have increased the sensitivity for mutation detection for EGFR in NSCLC, especially in cases with low levels of nucleic acids [254]. Joint analysis of ctRNA and ctDNA was highly sensitive to explore genomic alterations in metastatic castration-resistant prostate cancer in response to GT0918 (a potent AR antagonist) treatment [255]. Data indicate that a combined assay of CTC/ctDNA could enhance the sensitivity to diagnose primary lung cancer and may improve the early detection [60,61]. CTCs of the breast cancer might be able to predict the risk of CNS metastasis, since they show distinctive “breast cancer brain metastasis gene signature” [30]. It is not known yet, that ctDNA could provide such information. CTC may reveal the genetic signature of the tumor but shares significant overlaps with ctDNA. It seems that only a fraction of CTCs induces metastases [256]. Analyzing the tumor-derived exosome content may provide clinically useful information [257]. The tumor-derived exosomes may promote tumor progression by modulating the host micro-environment in a distant site. Since the tumor-derived exosomes preferentially fuse with resident cells at their predicted destination, the tumor exosome integrins may determine organotropic metastases [258]. There is data indicating that there are protein markers within the extracellular vesicles derived from breast cancer cells [259]. If this is proven in a clinical scenario for the solid tumors, that might further improve the utility of liquid biopsy.

The present standard for solid tumor evaluation is histopathological assessment of the obtained tumor tissue combined with imaging. However, frequently repeated biopsies for follow-up raise other problems such as the potential danger of biopsy (pain, bleeding, infection). Thus, replacement of biopsy with less-invasive interventions such as liquid biopsy is advantageous for the patients. Since the liquid-biopsy-obtained markers provide information for the tumor molecular phenotype, the combination of imaging and liquid biopsy might replace the present standard soon.

The liquid biopsy also makes it possible to obtain the serum protein tumor markers; thus, combined analysis of them with other liquid biopsy parameters is possible. Combined use of tumor markers and ctDNA obtained from cerebrospinal fluid was used in order to detect leptomeningeal metastases. The CSF cytology was positive 30.7%, whilst the MRI positivity was 58.9%. The sensitivity of CEA, NSE, and CFRA-211 was 75.8%, 51.7%, and 33.3% respectively. The ctDNA was positive in the CSF 92.3%, but the combined use of ctDNA and tumor markers obtained 100% positivity [260]. Combined analysis of pre-operative plasma ctDNA and protein tumor markers (CA 125 and CA 19-9) was carried out in a retrospective study. 19/51 patients without and 6/7 patients with detected ctDNA had recurrence (*p* = 0.001). 17/47 patients without and 8/10 with positive serum markers had recurrence (*p* = 0.0002). Altogether, 15 patients were positive for both ctDNA and serum markers and 12 of them had recurrence (*p* = 0.0001). Thus, the prediction of recurrence in surgically treated early-stage lung adenocarcinoma may be further improved by the combined analysis [261]. There are data available about the combined evaluation of the circulome and imaging. A study found that combined measuring the functional tumor volume (FTV) by MRI and ctDNA level might improve the prediction of both pathologic complete remission and recurrence risk in early breast cancer after neoadjuvant chemotherapy. In fact, ctDNA positivity after neoadjuvant chemotherapy contributed significantly to FTV in identifying patients who had an increased risk of metastatic recurrence or death (*p* = 0.004) [262]. In lung cancer patients, the decrease of ^meth^cfDNA between the baseline and the first control was in parallel with a decrease in CT-derived tumor surface area, independently from the tumor mutational status [263]. In 52 melanoma patients who received systematic therapy for metastatic disease, a comprehensive analysis was carried out regarding serial ctDNA and FDG-PET investigations. In a cohort study, it turned out that the ctDNA assay may not provide advantages for surveillance compared to imaging and CEA levels [264]. It was found that ctDNA is very useful as a complementary modality to functional imaging monitoring the tumor burden real-time, as well as genomic changes throughout the therapy [265]. As knowledge is increasing regarding all liquid-biopsy-obtained biomarkers (CTC, ctDNA, ctRNA, exosomes), in the near future, simultaneous analysis of these will be implemented in the personalized therapy of cancer [266]. The integration of all circulome biomarkers and their combinations, however, can only enter into the oncology practice if their use is justified by improved clinical outcomes [267]. Moreover, there is a lack of evidence that integration of liquid biopsy data can improve not only the outcome of therapy but also the quality of life of the patients

## 9. Conclusions

The decision of indicating adjuvant chemotherapy is still based on clinical risk stratification. However, among the clinically low-risk patients, more than 10%, depending on cancer type, will eventually relapse. Thus, more accurate stratification would be necessary. The cfDNA-based decision making has the potential of replacing clinical risk stratification in the future. In fact, liquid biopsy may become the primary method soon, not only for molecular profiling of cancer but also for selecting precision therapies. However, the histological structure of tumor tissue is needed, as well as the detection of protein expression including hormonal receptors that is only possible at present following tumor biopsy, but this cannot be carried out by investigating cfDNA [268]. Thus, one of the great potentials of liquid biopsy is the minimal risk of repeated sampling, especially if considered the wealth of information that could be extracted via the blood-based tumor compartment. The analysis of genetic changes and epigenetic alterations might be used for several other purposes such as screening for cancer, assessment for prognosis, evaluation of tumor burden, surveillance of recurrence, and monitoring treatment. If ctDNA monitoring could be implemented in medical oncology, it would also allow for the detection of molecular mechanisms driving the resistance. Since several different resistance alterations can coexist at different metastatic sites of the primary tumor, analysis of CTC or ctDNA may be an irreplaceable tool for detecting these collectively from a single sample of liquid biopsy. The analysis of liquid biopsy samples may also be a useful tool to find out the explanation of mixed clinical responses to therapy.

The usefulness of ctDNA mainly depends on how accurately it reflects the genetic changes detected in the tumor tissue sample. There are data indicating advantages and disadvantages as well. A clear advantage is that ctDNA may represent the whole tumor burden including metastases. Consequently, information regarding genetic alterations of the tumor mightbe more representative based on ctDNA than from tissue biopsy. Thus, clinical oncology decision making will be based on ctDNA in the future. There are data to prove that the application of ctDNA-based MRD analysis not only supports clinical decision making, but also enhances patient survival outcomes [269].

The disadvantage is the potential for non-concordance of key alterations. In fact, non-concordance is mainly observed in patients with low ctDNA levels. There are still many problems that need to be overcome such as improving the techniques of detection, especially considering the low amount of ctDNA and high background signals. Moreover, the ctDNA tests still have lower sensitivity to detect fusion events and copy number changes. Improvement of technologies is expected to allow more precise, quick, and inexpensive testing of ctDNA.

Integration of ctDNA in clinical decision making is inevitable but requires thorough understanding of the limitations, as well as usability to select effective therapies, detect resistance to therapy, or provide real-time monitoring of therapy response. It is also important to clarify the pros and cons of the techniques applied and the many details regarding the clinical application of these, e.g., optimal timing of liquid biopsy, the feasibility of sample processing in clinical circumstances, etc. The ongoing studies will answer these questions. Thus, standardization, as well as validation of the new platforms potentially applied in clinical practice is necessary before routine use. According to a clinically validated new platform for the next-generation sequencing of circulating tumor DNA point mutations, insertions/deletions, copy number alterations, gene fusions, as well as oncogenic viruses (Epstein–Barr virus (EBV) and hepatitis B virus (HBV)), and microsatellite instability (MSI) could be detected [79]. To fully integrate ctDNA tests in medical oncology, it is important to demonstrate that modifying therapy based on ctDNA changes before progression observed by radiology will improve overall survival in metastatic cases. The initiation of therapy after curative-intent therapy if the ctDNA test suggests MRD will provide benefit to the patients [78,198].

The other limitation of using ctDNA in oncology is that it is unable to detect non-genetic mechanisms of resistance. Moreover, it seems that ctDNA testing shows lower sensitivity than that available from tissue samples. Unfortunately, today, there is no single ctDNA test that could be used for all potential applications. Thus, to integrate ctDNA into routine care, it is necessary to improve sensitivity without a negative impact on specificity [270]. Since ctDNA is not always detectable in cancer patients, the use of ctDNA could not be implemented in the decision making for all patients. In certain cases (e.g., primary brain tumors, metastatic disease with brain metastases only), the amount of ctDNA in the blood is usually low; thus, the application of ctDNA tests are limited. However, using cerebrospinal fluid for the ctDNA test may overcome this problem [271]. Low ctDNA yield could also be expected in the case of nodal involvement, as well as in oligometastatic disease leading to false-negative results. The most uncertainty regarding the use of liquid biopsy exists in the case of early detection where it would be most useful. It is still an open question whether ctDNA is useful for diagnosing precancerous conditions in asymptomatic subjects.

The ctDNA is a powerful tool which has the potential to support tailored management of patients since it could support timely treatment decisions to optimize efficacy, it could provide real-time information regarding tumor activity, it is able to detect resistance, and minimize the unnecessary treatment burden for patients. In science, the promise of a new approach is not enough; only validated, cross-checked methodologies can be implemented in the use of patient care. However, if liquid biopsy meets the expectations of researchers in all areas investigated, it will revolutionize medical oncology [8]. The use of ctDNA demonstrated many potential benefits in the pre-clinical setting, but it is important that such benefits are confirmed in medical oncology where it should be established by prospective, randomized, and controlled clinical trials. Thus, further large-scale studies are needed to fine-tune the use of liquid biopsy in clinical oncology. In the recent ESMO guidelines on the use of ctDNA in clinical oncology, ctDNA was not recommended in routine clinical practice to detect molecular residual disease or molecular relapse [8]. The use of ctDNA is still under research development; thus, it is not recommended for routine clinical applications for identifying patients who are not responding to therapy based on early changes of ctDNA level, monitoring the development of resistance mutations before observing clinical progression, and screening for cancer in asymptomatic patients. The ctDNA assay, however, may be used to identify actionable mutations (e.g., testing for single nucleotide and small insertion and deletion variants) to direct targeted therapy if the limitations of the assay are considered.

All circulome biomarkers and their combinations will be integrated in routine oncology care if their use is justified by improved clinical outcomes. Moreover, there is a lack of evidence that integration of liquid biopsy data can improve not only the outcome of therapy such as the overall survival, but also the quality of life of the patients.

## Figures and Tables

**Table 1:** Comparison of CTC and ctDNA.

Aspect	ctDNA	CTC
Procedure	Minimally invasive	Minimally invasive
Sample collection	Prospective	Prospective
Intratumoral heterogeneity	Adequately represent, but false-negative and false-positive errors could occur more than 15% [5] May identify resistance mechanisms, discordant clinical history, and intertumor/intratumor heterogeneity	Adequately represent, but small and fragile sample population; detection increased with increasing stage
Phenotype [6]	Study the methylome may provide information about phenotype	Structural evaluation of the tumor might be possible
Tumor burden	Sensitive indicator	Still obscure how adequately represent tumor burden
Resistance	The emergence of resistance could be addressed	The emergence of resistance could be addressed
Recommended application NCCN and ESMO [7,8]	Should not be used to diagnose! Analysis DNA methylation changes, copy, and mutations can be used to identify EGFR, ALK, and other oncogenic biomarkers that would not otherwise be identified in patientswith metastatic cancer In ER+ breast cancer PIK3CA mutations to identify candidates for alpelisib plus fulvestrant In CRC, post-surgical ctDNA is a marker for an elevated risk of recurrence in stage I–III colon cancer	Should not be used to diagnose! May be considered at progression of NSCLC instead of tissue biopsy to detect whether patients have lung cancer; however, if plasma testing is negative, then tissue biopsy is recommended

## Abbreviations

5fC	5-formylcytosine
ALK	anaplastic lymphoma kinase gene
BRAF	human gene that encodes a protein called B-Raf
CA-125	cancer/ carcinoma antigen 125, or carbohydrate antigen 125
CEA	carcinoembryonic antigen
cfDNA	cell-free DNA
cfRNA	cell-free RNA
CHIP	clonal hematopoiesis of indeterminate potential
CI	confidence interval
CpG	dinucleotide DNA where a cytosine (C) nucleotide is followed by a guanine (G) nucleotide
CRC	colorectal cancer
CRPC	castrate-resistant prostate cancer
CSC	cancer stem cell
CSF	cerebrospinal fluid
CTC	circulating tumor cell
ctDNA	circulating tumor-derived DNA
DELFI	DNA evaluation of fragments for early interception
DFS	disease-free survival
DNA	deoxyribonucleic acid
EC	endometrial cancer
EGFR	epidermal growth factor receptor
ER	estrogen receptor
ESMO	European Society for Medical Oncology
ET	end of treatment
EV	extracellular vesicle
FDA	Food and Drug Administration
FTV	functional tumor volume
HER2	human epidermal growth factor receptor 2
HR	hazard ratio
ICB	immune checkpoint blockade
KRAS	“Kirsten rat sarcoma virus” is a gene that provides instructions for making a protein called K-Ras
LB	liquid biopsy
mAb	monoclonal antibody
MRD	minimal residual disease
mRNA	messenger RNA
MSI	microsatellite instability
MT	middle of thetherapy
mtDNA	mitochondrial DNA
NAT	neoadjuvant therapy
NCCN	National Comprehensive Cancer Network
NCDB	National Cancer Database
NGS	next-generation sequencing
NSCLC	non-small-cell lung cancer
OC	ovarian cancer
OR	odds ratio
OS	overall survival
PCR	polymerase chain reaction
PFS	progression-free survival
PIK3CA	phosphatidylinositol-4,5-bisphosphate 3-kinase catalytic subunit alpha
PSA	prostate-specific antigen
RFS	relapse-free survival
RNA	ribonucleic acid
RT	radiotherapy
SCLC	small cell lung cancer
TEP	tumor-educated platelets
TET	ten-eleven translocation enzyme
TKI	tyrosine kinase inhibitor
TMB	tumor mutational burden
TNBC	triple-negative breast cancer
TP53	tumor protein p53
VAF	variant allele frequency
